# Altered Secretome and ROS Production in Olfactory Mucosa Stem Cells Derived from Friedreich’s Ataxia Patients

**DOI:** 10.3390/ijms21186662

**Published:** 2020-09-11

**Authors:** Sara Pérez-Luz, Frida Loria, Yurika Katsu-Jiménez, Daniel Oberdoerfer, Oscar-Li Yang, Filip Lim, José Luis Muñoz-Blanco, Javier Díaz-Nido

**Affiliations:** 1 Centro de Biología Molecular Severo Ochoa (CSIC-UAM) and Departamento de Biología Molecular, Universidad Autónoma de Madrid, Nicolás Cabrera, 1, 28049 Madrid, Spain; sara.perez@isciii.es (S.P.-L.); daniel.ober@gmail.com (D.O.); qoscaryang@gmail.com (O.-L.Y.); javier.diaznido@uam.es (J.D.-N.); 2 Molecular Genetics Unit, Institute of Rare Diseases Research, Institute of Health Carlos III (ISCIII), Ctra. Majadahonda-Pozuelo Km 2,200, 28220 Madrid, Spain; 3 Laboratorio de Apoyo a la Investigación, Hospital Universitario Fundación Alcorcón, Calle Budapest 1, 28922 Madrid, Spain; 4 Karolinska Institutet, Department of Microbiology Tumor and Cell Biology, Solnaväjen 1, 171 77 Stockholm, Sweden; yurika.katsu@ki.se; 5 Department of Molecular Biology, Autonomous University of Madrid, Francisco Tomás y Valiente 7, 28049 Madrid, Spain; filip.lim@uam.es; 6 Department of Neurology, Hospital Universitario Gregorio Marañón, Dr. Esquerdo 46, 28007 Madrid, Spain; jmunozb@salud.madrid.org

**Keywords:** Frataxin, Friedreich´s ataxia, gene therapy, stem cells human olfactory mucosa

## Abstract

Friedreich’s ataxia is the most common hereditary ataxia for which there is no cure or approved treatment at present. However, therapeutic developments based on the understanding of pathological mechanisms underlying the disease have advanced considerably, with the implementation of cellular models that mimic the disease playing a crucial role. Human olfactory ecto-mesenchymal stem cells represent a novel model that could prove useful due to their accessibility and neurogenic capacity. Here, we isolated and cultured these stem cells from Friedreich´s ataxia patients and healthy donors, characterizing their phenotype and describing disease-specific features such as reduced cell viability, impaired aconitase activity, increased ROS production and the release of cytokines involved in neuroinflammation. Importantly, we observed a positive effect on patient-derived cells, when frataxin levels were restored, confirming the utility of this in vitro model to study the disease. This model will improve our understanding of Friedreich´s ataxia pathogenesis and will help in developing rationally designed therapeutic strategies.

## 1. Introduction

Friedreich’s ataxia (FRDA, OMIM229300), the most common hereditary ataxia among Caucasians, is predominantly a neurodegenerative disease affecting the dorsal root ganglia, spinal cord, brainstem and cerebellum [1], with extra-neurological manifestations such as hypertrophic cardiomyopathy [2] and diabetes mellitus [3]. FRDA is an autosomal recessive genetic disease caused by mutations in the *FXN* gene (MIM 606829), which encodes the mitochondrial protein frataxin [4]. The majority of FRDA patients have an abnormal expansion of the triplet GAA inside the first intron of the *FXN* gene [5]. This expansion causes a reduction in the expression of the protein [6,7] by formation of a non-B DNA structure, persistent RNA–DNA hybrids or heterochromatin formation [8]. Frataxin is synthesized as a precursor form, which is subsequently imported to the mitochondria, where it undergoes consecutive proteolytic cleavages by the mitochondrial processing peptidase (MPP) to produce the mature form [9]. There are different biological functions in which frataxin plays an important role, including iron homeostasis, iron–sulphur cluster biosynthesis, modulation of oxidative phosphorylation and regulation of the response to oxidative stress. Nevertheless, it is still not known how frataxin deficiency triggers the neurodegenerative process associated with the disease (for a review of frataxin function, see [10,11]).

Currently, there is no cure or efficient treatment for FRDA and disease management is focused in ameliorating the physical symptoms associated with its progression. Several therapeutic approaches to arrest and/or slow down the disease are under development and can be grouped into those aimed at improving mitochondrial function and reducing oxidative stress, those trying to increase or stabilize frataxin levels, and gene therapy (for a review of FRDA therapeutic approaches, see [12]). Other emerging and promising therapies include stem cell therapy [13], genome editing [14], and oligonucleotide-based approaches [15]. As FRDA is a monogenic loss-of-function disease, it is an ideal candidate for gene therapy, as introducing a healthy copy of the gene is predicted to rescue the disease phenotype [16,17]. However, some issues remain to be solved such as avoiding toxicity of *FXN* over-expression, ensuring safety of vectors used, or how to specifically reach deeply buried cells of the central nervous system, the main tissue affected [4]. Facilitating the advance of all such aspects of therapeutic development, with the availability of appropriate cellular models that closely mimic the disease, is a high priority.

Different cell models are used to study the molecular pathogenic mechanisms implicated in FRDA, but given the neurodegenerative nature of the disease, the use of neural cell models that mimic FRDA in a dish, is particularly relevant [18]. Frataxin deficiency has been induced in a variety of rodent and human neural cells by RNA interference [19,20], but the generation of stable cell models using this approach is difficult as *FXN* gene knockdown increases cell death and interferes with long-term proliferation. To avoid this hindrance, a different approach has been implemented by using patient-derived cell lines, which already have reduced levels of the protein. In this sense, the most widely used cells have been fibroblasts and blood-derived lymphoblasts, as they are more readily accessible [21,22,23]. However, they are non-neuronal cells and accordingly, may lack important features necessary for understanding the cellular and molecular basis of neurological diseases like FRDA. In addition, induced pluripotent stem cells (iPSCs) obtained by genetic reprogramming of patient-derived fibroblasts have also been generated as FRDA cell models, since they can be differentiated into key cell targets like neurons or cardiomyocytes [14,18,24]. However, this reprogramming might have secondary effects in the cells, possibly making them less representative of the disease as cell models [25].

Several studies indicate that human olfactory mucosa may be another accessible tissue to culture stem cells with a neurogenic potential [26,27], as biopsies of the human olfactory mucosa are quite easy to obtain with minimally invasive procedures, which generally lack significant side effects [28,29]. The olfactory mucosa, responsible for the sense of smell, is a bi-stratum region containing stem cells with neurogenic capacity [30,31]. At least two types of human mucosa stem cells have been described: cytokeratin-positive neuroepithelial stem cells, which are located in the olfactory epithelium, and Stro-1-positive olfactory ecto-mesenchymal stem cells (OE-MSCs), from the lamina propria [32,33]. The utility of neuroepithelial stem cells to model brain disorders has been documented elsewhere [34].

On the other hand, OE-MSCs have been described as being similar to the well characterized mesenchymal stem cells (MSCs) [35]. However, they exhibit specific features such as high proliferative rate with little or no apoptotic activity, potential for osteogenesis and poor chondrogenic and adipogenic potential [33,36]. Within the olfactory system, these cells can play a reparative role after extensive peripheral damage, as they are able to cross the basement membrane in order to differentiate into neurons and replace the olfactory epithelium if necessary [33], in addition to their previously demonstrated capacity to promote myelination [37]. Some groups have differentiated OE-MSCs into different lineages [38,39] and have even shown their regenerative properties in several models of neurodegenerative disorders such as Parkinson´s disease [40] or spinal cord injury [41]. Recently, OE-MSCs were differentiated into dopaminergic neurons [42] and motor neuron-like cells [43]. However, in spite of being considered as neural stem cells [44], a complete characterization of their in vitro neuronal differentiation potential is still missing, and studies addressing the existence of disease-relevant characteristics in cells obtained from patients with neurodegenerative diseases have lagged behind.

In this work, we investigated the phenotype of human olfactory mucosa stem cells derived from healthy donors and FRDA patients, aiming to characterize them and to test whether they might serve as an in vitro model to study the etiopathogenesis of FRDA. We confirmed by immunofluorescence and RT-PCR that cells derived from biopsies from a healthy donor and two patients have the markers corresponding to OE-MSCs. FRDA-derived cells exhibited reduced frataxin and aconitase levels that were accompanied by increased ROS production, changes in their secretome and higher cell death rates than cells derived from a healthy donor. Furthermore, we were able to partially rescue this phenotype by increasing frataxin levels, which confirms the utility of this in vitro approach for modeling the disease.

## 2. Results

### 2.1. Olfactory Mucosa Stem Cells Isolated from Biopsies Exhibit a Phenotype Corresponding to Neural Crest-Derived Mesenchymal Stem Cells

After obtaining the biopsies, dissociated cells were plated and maintained in CSC medium for 2–3 days, after which cells started to attach and proliferate. Initially, cells presented a low proliferative rate that was enhanced by modifying culture conditions with the addition of FBS (2%) and growth factors (NGF and rhFGF2), and by culturing them with Matrigel. We evaluated frataxin expression in the different isolated cell populations from FRDA patients and healthy donors. Of the seven cells lines tested (three healthy donors and four FRDA patients) we decided to keep on working only with those cell lines showing a balance between their proliferative rates and their frataxin levels.

**Figure 1:**
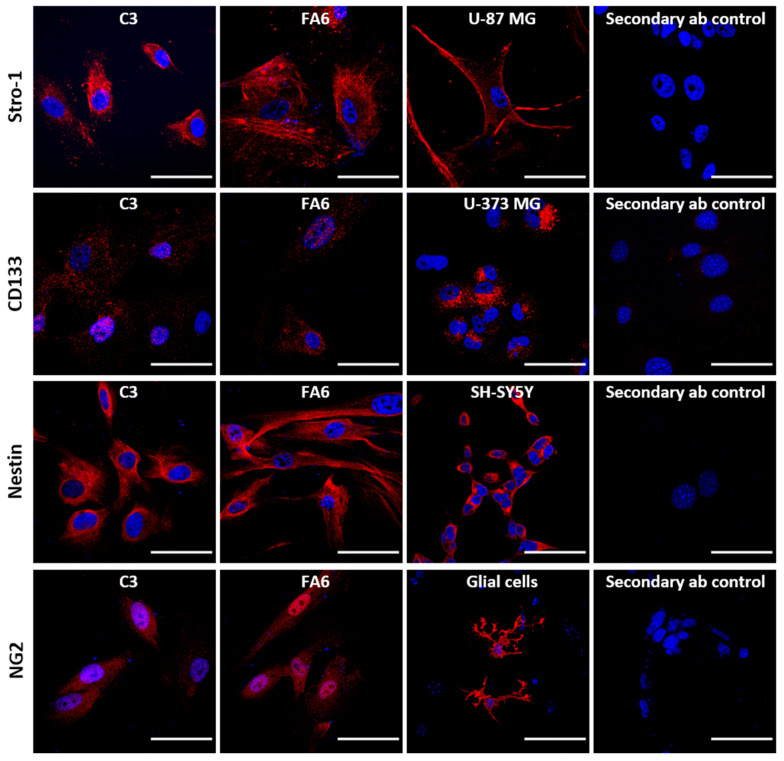
OE-MSC immunocytochemical characterization. Representative immunofluorescence confocal photomicrographs of mucosa-derived mesenchymal stem cells (C3 and FA6) labelled in red with Stro-1, CD133, Nestin and NG2. Nuclei were stained with DAPI (blue). Scale bars = 50 μm.

**Figure 2:**
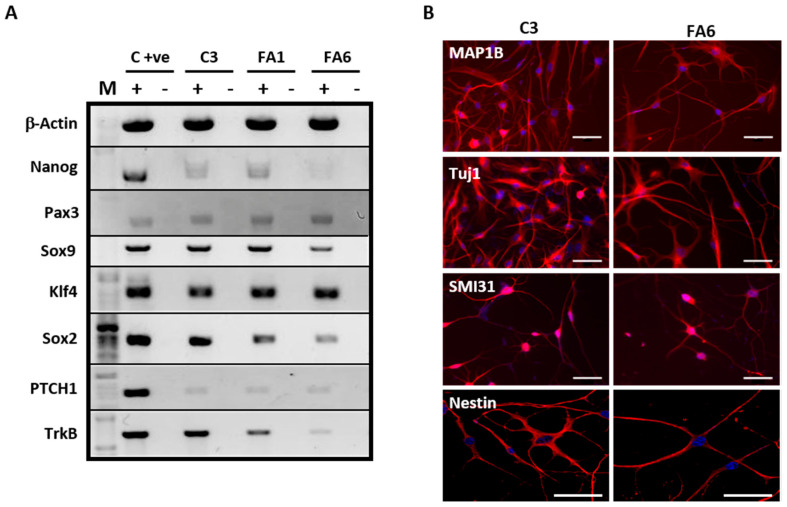
OE-MSC expression of different pluripotent and stem cell markers. (**A**) RT-PCR showing the expression of different markers for stemness and pluripotency in OE-MSCs. Expression of the housekeeping gene for β-Actin was used as a reference control. M: 1Kb ladder, C+ve: PCR reaction control using hESC H9 cell line (Nanog, Klf4, and Sox2) and neuronally differentiated SH-SY5Y cells (Sox9, Pax3, PTCH1 and TrkB), (+): cDNA used as template for PCR amplified with RT enzyme, (−): no RT enzyme. (**B**) Immunophenotyping of C3 and FA6 samples showed these cells are positive (red) when stained with antibodies specific for MAP1B, Tuj1, SMI31 and Nestin. DAPI staining is shown in blue. Scale bars = 50 μm.

To characterize the isolated cells, immunophenotyping was carried out after several passages of cells derived from control cells (C3) and FRDA-derived cells (FA1) using different antibodies to determine stemness (Figure 1). Cells were positive for Stro-1, one of the best-known markers of MSCs [32], and for Nestin and NG2, two neural stem cell markers, already described as exhibited by this type of mucosa-derived cells as they originate from the neural crest [33]. Other stem cell precursor markers such as CD133, exhibited by neural stem cells [45], are expressed by the population isolated albeit less strongly, although we could not confirm the expression of CD49d, typically expressed by MSCs [46]. Importantly, all cells were negative for cytokeratin (data not shown). We further characterized the phenotype of the olfactory mucosa samples by evaluating mRNA expression of other markers by RT-PCR (Figure 2A).

Cells isolated from the human olfactory mucosa of controls and FRDA patients were positive for Nanog, an embryonic stem cell marker [47] and Klf4, a pluripotent stem cell marker [48]. The samples also expressed Sox9, an early neural crest derived marker [49], Sox2, required for stem-cell maintenance in the central nervous system [50] and Pax3, involved in neural development [51]. In addition, mucosa-derived cells expressed TrkB, a receptor of the neurotrophin BDNF able to mediate proliferation and differentiation of neuronal cells [52] and PTCH1, a transmembrane receptor in the Sonic-Hedgehog pathway, also found to be expressed in embryonic stem cells [53]. Overall, we found that the phenotype of both control and FRDA olfactory mucosa samples corresponds to OE-MSCs of the lamina propria. To further confirm the stemnness of these cells, we performed a functional characterization by testing their capacity to differentiate into neural cells. With the two-step differentiation protocol, the cells stopped proliferating and showed a complex morphology after 2 days in the second medium. The immunocharacterization of OE-MSCs shows neuronal commitment after 8 days of differentiation (Figure 2B), demonstrated by reactivity using neuronal cytoskeleton-specific antibodies MAP1B, Tuj-1 and SMI31, along the neuron-like extensions and the cell body. However, the presence of positive nestin staining, a neuronal stem cell marker, was still observed. These results indicate that OE-MSCs from healthy donors and FRDA patients can differentiate into neuron-like cells, but they retain some features of neural stem cells.

### 2.2. OE-MSCs from FRDA Patients Express Low Frataxin Levels, Exhibit Increased ROS Production and Decreased Cell Viability

**Figure 3:**
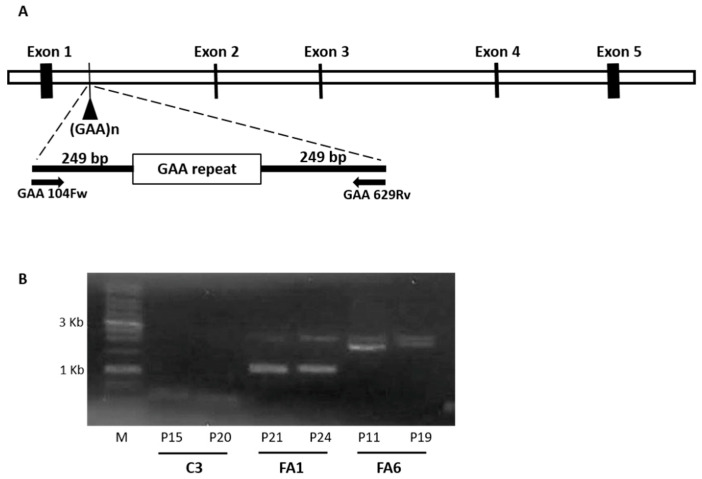
Number of GAA repeats in FRDA-derived OE-MSCs. (**A**) Schematic representation of the location of the primers used for the PCR. (**B**) Gel showing the number of GAA trinucleotide repeat in the different cell lines. PCR was carried out at two different passages (P) to check for in vitro GAA variations. C3 samples were analyzed at passage 15 and 20 (P15 and P20, respectively), FA1 at passage 21 and 24 (P21 and P24, respectively), and FA6 at passage 11 and 19 (P11 and P19, respectively). M: 1 kb ladder (Biotools), C3: cells derived from a healthy donor, FA1 and FA6: cells derived from FRDA patients.

Friedreich´s ataxia is mainly caused by a GAA trinucleotide repeat expansion within the first intron of the *FXN* gene [5]. To determine the number of such expansions in the OE-MSCs, we performed a PCR assay as described by Campuzano et al. [5] at two different culture passages. C3 cells showed a PCR product of approximately 560 bp, equivalent to 14 GAA repeats as the amplified PCR product included the trinucleotide expansion with flanking sequences of 249 bp at both the 5′ and 3′ end (Figure 3A,B). In contrast, both FRDA-derived OE-MSCs rendered heterozygous longer PCR amplification products, with FA1 presenting two amplification bands of 1200 and 2000 bp (~230 and ~500 GAA repeats, respectively) and FA6 showing two longer amplification bands of 2000 and 2500 bp (~500 and ~670 GAA repeats, respectively), both in the range of pathogenic alleles, which are between 66 and 1700 repeats [54]. We did not observe differences in GAA repeat expansions with different passage numbers of any of the cultures of C3, FA1 or FA6 cells.

**Figure 4:**
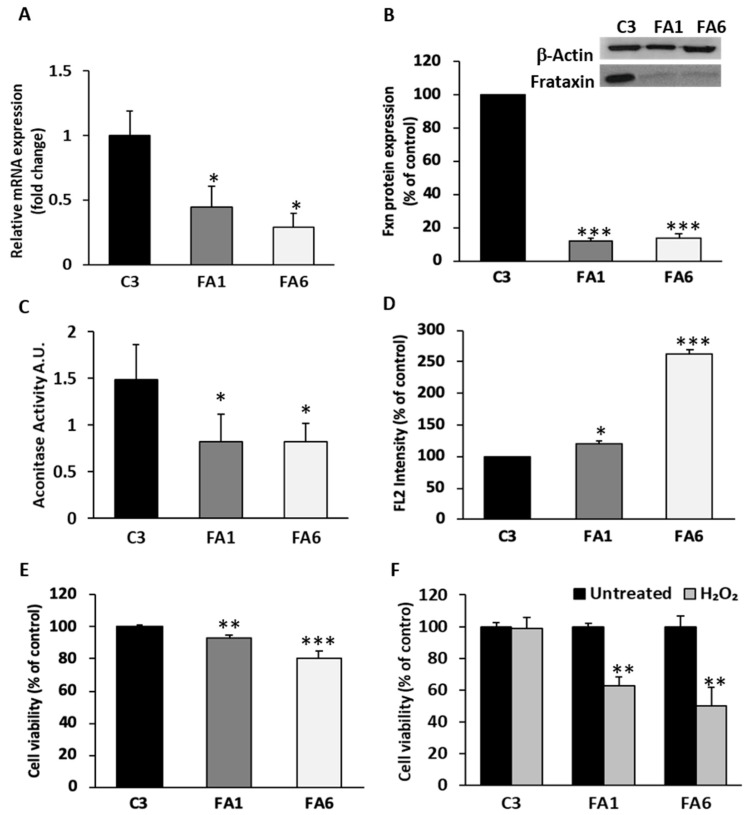
FRDA-patient-derived OE-MSCs phenotype. (**A**) Quantification of frataxin mRNA expression in the three cell lines. FA1 and F6 frataxin levels are expressed relative to control levels (C3). Values were normalized to β-actin and the histogram represents the mean fold change ± SEM from three independent experiments. (**B**) Representative Western blot showing frataxin expression in OE-MSCs. The bar graph shows the corresponding quantification of values normalized to β-actin. Values are expressed as mean ± SEM relative to C3 cells. * *p* < 0.05; *** *p* < 0.005 compared to control. (**C**) Bar graph showing aconitase activity assessed in the three samples of mucosa-derived OE-MSCs. The activity of aconitase enzyme in FRDA patients (FA1 and FA6) is almost reduced to half its normal activity compared with control cells (C3). (**D**) Quantification of ROS levels by flow cytometry using the fluorophore MitoSOX also revealed an abnormal production of ROS in patient-derived cells, which is quite elevated in FA6 cells. (**E**) Viability measurements in OE-MSCs show a reduced number of patient-derived cells (light and dark grey bars) relative to control cells (black bar). (**F**) Percentage of cell survival in C3, FA1 and FA6 cells after 24 h of H_2_O_2_ treatment (dark grey). In (**A**–**E**), data represent the mean ± SEM from three independent experiments. * *p* < 0.05; ** *p* < 0.01; *** *p* < 0.001 vs. C3. In (**F**), ** *p* < 0.001 vs. Untreated FA1 and FA6, respectively.

We then determined frataxin mRNA expression by qPCR and found that both FA1 and FA6 OE-MSCs have significantly less frataxin levels than C3 control cells (Figure 4A). Our results showed that FRDA-derived OE-MSCs have less than half the mRNA levels of unaffected individuals. Next, we evaluated frataxin protein levels by Western blotting, since it is the amount of the mature protein that determines the severity of the disease. Again, patient-derived OE-MSCs had significantly lower levels of the mature protein when compared to C3 control cells (Figure 4B). The relative percentage of frataxin expression obtained by densitometric analysis of FA1 and FA6 cells showed that mature frataxin expression is reduced to less than 20% in both cases, relative to healthy donor levels.

Aconitase is an iron–sulphur protein implicated in both the tricarboxylic citric acid (mitochondrial isoforms) and glyoxylate cycles (cytosolic isoforms), containing a (4Fe-4S) cluster [55]. It has been shown that frataxin is actively implicated in the first step of the Fe-S cluster biogenesis, supervising iron entry into the emerging Fe-S cluster [56]. As aconitase is one of the first enzymes affected by frataxin deficiency, we evaluated its enzymatic activity in OE-MSCs and observed that it was reduced to almost 50% of normal levels in FRDA-derived OE-MSCs (Figure 4C). Another characteristic feature of FRDA patient cells is the increased levels of oxidative damage due to a rise in the production of free radicals [57]. We therefore evaluated the levels of reactive oxygen species (ROS) in mucosa-derived cell lines, using flow cytometry and the fluorescent probe MitoSOX Red, a measure of mitochondrial superoxide production (Figure 4D). Quantification of ROS production in FA1 and FA6 cells showed a significant increase in ROS production in both cell lines, although FA6 cells exhibited a more dramatic boost, almost three times higher than healthy cells.

Frataxin-deficient cells are also characterized by their increased susceptibility to oxidative stress [58], which can in turn cause cell death. Using the calcein/propidium iodide viability assay, we quantified the proportion of live/dead cells in OE-MSCs, and verified that basal levels of cell death are higher in frataxin-deficient cells than in control cells (Figure 4E), which could be attributed to the shortage of frataxin protein in FRDA-derived OE-MSCs. To determine OE-MSC sensitivity to oxidative stress, C3, FA1 and FA6 cells were exposed to 100 µM H_2_O_2_ for 24 h, after which we measured cell viability. While control OE-MSCs tolerated H_2_O_2_ treatment without significant changes in cell survival, FRDA patient-derived cells subjected to this oxidative insult exhibited a dramatic drop in cell viability, decreasing to almost 50% of untreated cell numbers (Figure 4F).

### 2.3. Aconitase Activity is Restored in Patient-Derived OE-MSCs Transduced with Lentivirus Encoding for Frataxin

**Figure 5:**
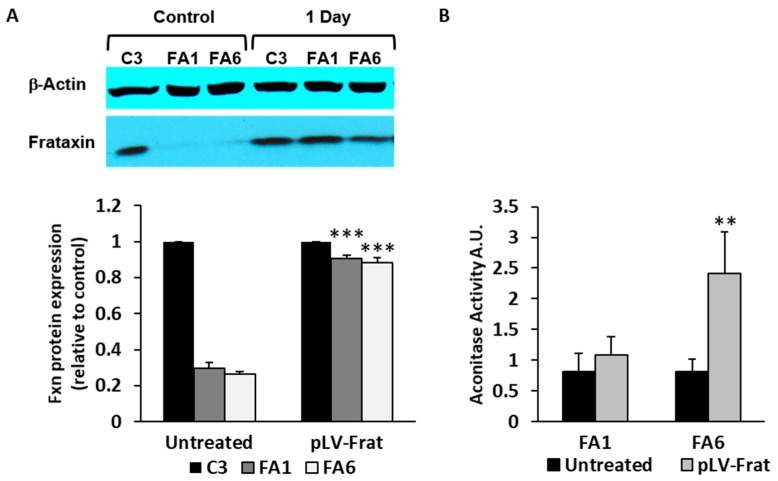
Frataxin rescue in OE-MSCs revert the abnormal phenotype. (**A**) Representative Western blot of OE-MSCs transduced for 24 h with pLV-Frat, a lentivector encoding a cDNA for frataxin. The bottom left graph shows the quantification after normalization to β-actin and densitometry. Values are expressed as mean ± SEM and cells are compared with their respective non-transduced (untreated) control. *** *p* < 0.005. (**B**) Aconitase levels (arbitrary units) in FA1 and FA6 cells, after 24 h of frataxin overexpression. Data represent the mean ± SEM of three independent experiments. ** *p* < 0.005 compared with non-transduced FA6.

Once the OE-MSC samples had been characterized, we investigated whether frataxin overexpression could restore some of the phenotypic features already described. To do this, control and FRDA-patient-derived cells were transduced with a lentiviral vector (pLV-Frat) encoding a human frataxin cDNA, which was previously characterized in our laboratory [19]. In all cell lines (C3, FA1 and FA6), we used a MOI of 10 of pLV-frat to overexpress frataxin to similar levels as those observed in control cells (C3). Using this MOI, we achieved a transduction efficiency of ~80% after 24 h. Western blotting analysis demonstrated that frataxin levels in C3 cells did not change significantly at 24 h post-transduction. In contrast, when compared to non-transduced cells, frataxin levels in FA1 and FA6 cells were significantly higher after pLV-frat transduction. Indeed, mature frataxin levels were almost completely restored to control values (C3), presumably compensating the lack of the protein in those cells (Figure 5A).

As a biomarker of recovery of the normal phenotype of frataxin-deficient cells after lentiviral transduction, we measured aconitase activity. Aconitase enzymatic activity measured in FA1 and FA6 cells transduced with pLV-Frat for 24 h showed increased levels, especially in FA6 cells (Figure 5B), in agreement with previous studies and highlighting the close relationship between frataxin and aconitase [59,60,61].

### 2.4. Up-regulation of Pro-Inflammatory Cytokines in Friedreich´s Ataxia Patient-Derived OE-MSCs

**Figure 6:**
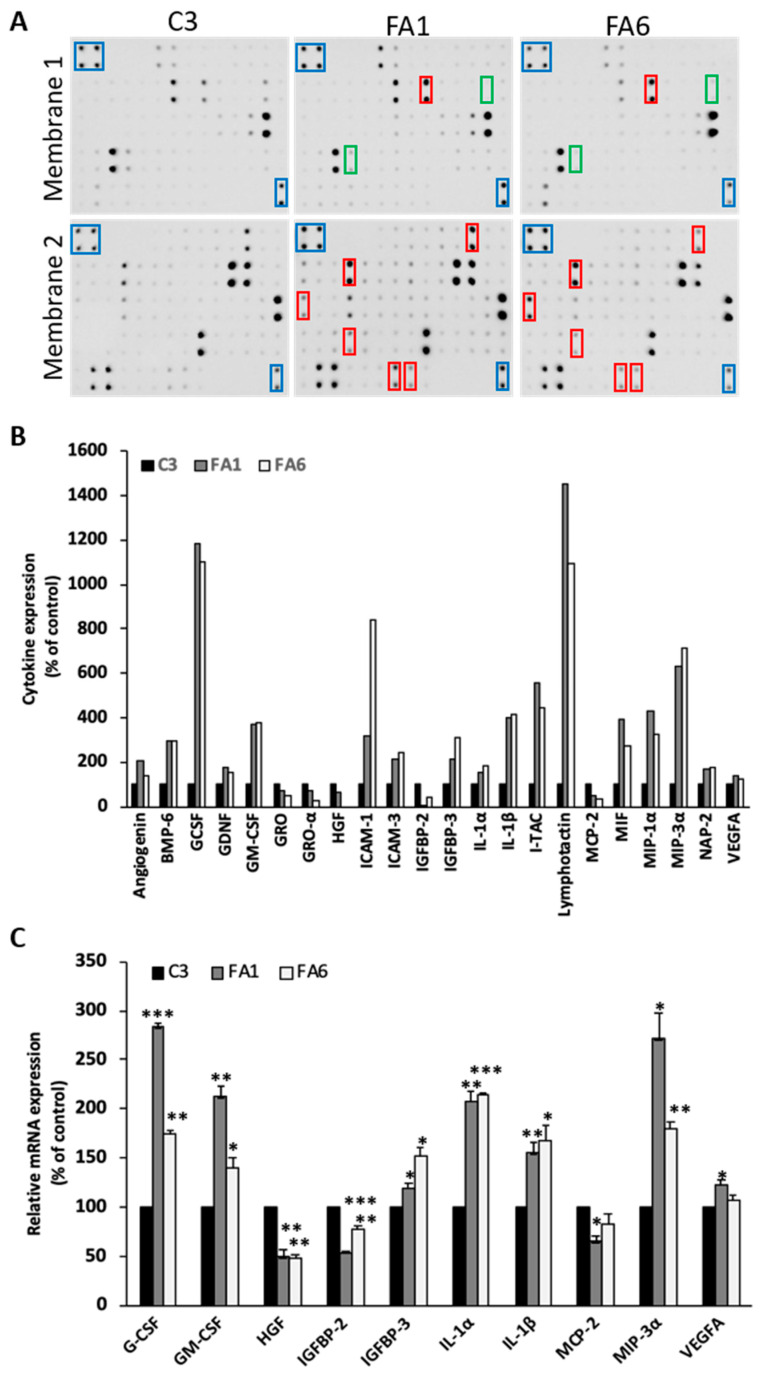
Cytokine profile of FRDA patient-derived OE-MSCs. (**A**) Representative antibody-based membrane arrays incubated with serum-free medium from C3, FA1 and FA6 OE-MSCs. Rectangles indicate positive controls (blue), some upregulated cytokines (red) and some downregulated cytokines (green). (**B**) Bar graph showing the quantification of selected up-regulated and downregulated cytokines released by the three cell lines and normalized to C3 levels. (**C**) Q-PCR amplification of selected cytokines. mRNAs from the different OE-MSCs were quantified using the Ct method. Values are expressed as mean ± SEM in relative percentage of control cells from three independent samples. * *p* < 0.05; ** *p* < 0.005; *** *p* < 0.001 compared with C3.

To characterize in more detail the phenotype of FRDA olfactory mucosa samples, we analyzed the secretome of C3, FA1 and FA6 cells. Serum-free medium of 24 h was collected and analyzed using a protein-based array that identifies up to 120 cytokines (Figure 6A). When compared with control OE-MSCs, we observed increased secretion of 27 cytokines and decreased secretion of 8 cytokines in FRDA-derived cells (Figure 6B showing the more relevant findings). According to their principal functions, these cytokines were classified into the following groups: (a) angiogenesis/proliferation, (b) iron regulating proteins, (c) immunity and inflammation, (d) neurotrophic factors, (e) growth factors, and (f) growth factor binding proteins (Table 1). With the aim of validating these arrays, qPCR was performed to verify the mRNA levels of the cytokines differentially expressed in cells from FRDA patients. Results of cytokines validated at the transcriptional level and compared with C3 cells, in FA1 and FA6 cells, are shown in Figure 6C. mRNA expression of IL-1α, IL-1β, MIP-3α, GM-CSF, G-CSF, and IGFBP-3 was significantly higher in frataxin-deficient cells, with VEGF-α presenting a clear but modest increase in both patient cell lines. On the other hand, among the downregulated cytokines of the array that also had significantly decreased mRNA levels were HGF, MCP-2 and IGFBP-2. In addition, we found several genes (GRO-α, SDF-1, KITLG, TGFB-1, and NAP-2) in which mRNA expression levels did not correspond to the cytokine array data, probably indicating that other post-transcriptional regulatory events could have taken place, increasing cytokine secretion and leaving mRNA levels intact.

## 3. Discussion

### 3.1. Olfactory Stem Cells Isolated from FRDA Patients Share Features Corresponding to OE-MSCs

FRDA is a highly disabling pathology lacking effective treatment to limit or stop its progression. To better understand the pathological mechanisms underlying the disease, there is a need to develop research models that are able to faithfully mimic various key features of this complex malady. Among the different benefits of using cells derived from healthy controls and FRDA patients, is that such models would reflect biological variations that are inherent in sampling from different individuals. In this sense, the potential use of olfactory-derived mucosa stem cells to model neurogenetic and neurodegenerative diseases like FRDA is promising, as these cells are easily obtained by a non-invasive biopsy, and grow well in culture.

Human stem cells from the olfactory mucosa have been described before as originating from both the neuroepithelium and the lamina propria [32,33]. Neuroepithelial stem cells have been more extensively studied and have been described as a useful model to recapitulate and study several neurodegenerative diseases such as ataxia-telangiectasia [62]. However, stem cells from the other olfactory mucosa stratum have been less investigated. The possible function of these lamina propria-derived stem cells may be the recovery of the olfactory tissue when injured, including migration and differentiation into different cell types that form the mucosa. Despite being in a neurogenic niche, transcriptome profile characterization determined that these cells can be considered as MSCs with ectodermal characteristics, later named as OE-MSCs [33]. Here, we report the isolation and characterization of stem cells obtained from the lamina propria of olfactory mucosa of FRDA patients and healthy subjects. We have tested different protocols to culture these cells, and have optimized the procedure in terms of growth rate and stable maintenance over many passages, especially for patient-derived cells. In agreement with data obtained by Delorme and collaborators [33], the results of our immunophenotyping showed that these cells closely resemble MSCs, which are known for their pluripotent potential [63].

Our mucosa-derived cells have a high proliferative activity and can be maintained in culture long-term without any cell alterations, independently of their phenotype (FRDA-derived or healthy subjects, data not shown). To verify the double feature of these cells (meso- and ectodermal characteristics), we carried out immunophenotyping using a series of specific markers for both cell types. Cells were positive for neuronal progenitor markers (Nestin, NG2 and CD133) and the stromal progenitor markers Stro-1. CD133 is a marker of neuronal progenitor cells [64] as well as Nestin, a marker of neuronal precursor cells from the subventricular zone [65]. NG2 is present in cells that are able to convert to neurons in vitro [66] and in vivo [67], conferring responsiveness to CNS injury, and the ability to regenerate oligodendrocytes [68]. Stro-1 is considered the major MSC marker, a cell surface protein that is expressed by bone marrow stromal cells and erythroid precursors [69,70]. On the contrary, our cells were not positive for the MSC marker CD49d [71], an integrin-α subunit that can interact with Paxillin and LGALS8, two signal transduction proteins related to proliferation and differentiation, respectively [72,73]. In general, the immunophenotypic profile observed in our isolated cells suggests that these cells correspond to OE-MSCs [33]. Furthermore, we also found that cells derived from healthy subjects and FRDA patients were positive for other markers of pluripotent stem cells and some elements of signaling pathways involved in early development, with no clear difference between patient and control cells.

### 3.2. Phenotype of FRDA-Derived Olfactory Mucosa Cells

The hallmark feature of FRDA consists of a GAA repeat expansion of variable length, which impairs transcription of the *FXN* gene [74,75]. This variation in the number of repeats is closely linked to disease onset and severity. When we analyzed the number of GAA repeats in OE-MSCs from two FRDA patients, we found a dramatic increase in the repeat number (evidenced by longer PCR amplification products), compared to cells from a healthy subject. Indeed, we detected that FA6 cells have a higher GAA repeat number than FA1 cells, which could explain differences in some of the parameters measured in our experiments, between these two cell populations. Furthermore, we found that GAA repeat length is inversely related to frataxin mRNA levels in FRDA-derived OE-MSCs, with lower RNA levels found in FA6 cells, compared with FA1 cells. However, despite the fact that FRDA-derived cells had significantly lower frataxin protein expression levels than control cells, we did not find significant differences between patient-derived cells. This could mean that more repeat numbers are necessary to cause higher differences in protein levels between the two cell groups.

The main characteristic of frataxin deficiency is a high sensitivity to oxidative stress caused by the impairment of iron homeostasis, which varies depending on the cell type. In this study, we detected that FRDA patient-derived OE-MSCs had an impaired basal activity of the enzyme aconitase. This agrees with previous data indicating that frataxin is essential in the maturation of aconitase as an iron metabolism regulatory enzyme [76]. In addition, we also found that FRDA-derived cells had a higher rate of mitochondrial superoxide production than cells obtained from healthy subjects, with FA6 cells showing the highest levels. These results may reflect that a lack of frataxin does not only cause mitochondrial dysfunction in these cells, but also that mitochondrial aconitase function is affected, features that have been previously observed in FRDA and other neurodegenerative diseases [77]. Moreover, we determined that controls cells have higher survival rates than patient-derived cells; indeed, cell survival was even more affected in FRDA-derived cells than in control-derived cells, when cultures were exposed to the well-known oxidative stress inducer H_2_O_2_.

Among the strategies that have been tested as potential treatments for FRDA, is the restoration of frataxin levels and function using different approaches. We found that transducing FRDA patient-derived OE-MSCs with a lentiviral vector encoding for human frataxin, restored not only frataxin protein levels in patient-derived cells, but also aconitase activity. We consider these results as proof of concept of the validity of the use of OE-MSCs as a useful in vitro model of FRDA, in spite of the small number of samples included in our analyses. Whether these cells can be successfully differentiated to other CNS cell types and used in therapeutic approaches, as has been proposed for other diseases [78], still needs to be experimentally determined.

### 3.3. Cytokine Profile of FRDA-Derived OE-MSCs

We characterized the secretome of patient-derived OE-MSCs and healthy controls in an attempt to further understand the physiopathology of the disease and to identify novel therapeutic targets. From this study, we observed that the release of soluble factors and mRNA expression levels of many cytokines was significantly increased, while that of others was significantly decreased in FRDA-derived cells. However, some of the results obtained from the array are not consistent with the qPCR results. This negative correlation could be attributed to primers not being specific to the exact transcript of the gene of interest, or even due to posttranslational modifications that increase cytokine secretion but leave mRNA levels intact. Based on the consistency of results between array and qPCR, the most relevant results obtained from this study were the increased expression of IL-1α and IL-1β in patient cells, which agrees with previous results [79], as well as the increase in MIP-3α and decrease in HGF. IL-1α and IL-1β have been identified as potent pro-inflammatory cytokines in many neurodegenerative diseases such as Alzheimer’s disease, Parkinson’s disease or multiple sclerosis [80], and have been suggested to accelerate the progression of neurodegeneration. Furthermore, MIP-3α is a chemokine that has been associated with neurodegeneration [81,82]. Since these cytokines are upregulated at the mRNA and protein levels, this strongly suggests that an inflammatory process is active in these OE-MSCs from FRDA patients. On the other hand, HGF is known to be a potential neurotrophic factor, and has been shown to diminish Purkinje cell degeneration in spinocerebellar ataxia type 7 when overexpressed [83]. Besides, it has antiapoptotic activities by inhibiting caspase-3 [84]. A reduction in both secretion and mRNA expression of HGF could suggest that both apoptosis and cell degeneration are enhanced in FRDA patient-derived cells.

In addition, the results obtained from our human cytokine array of OE-MSCs are consistent with the results obtained from the array of frataxin-deficient Schwann cells published by Lu and collaborators [79], where IL-6, IL-1α, G-CSF, GM-CSF, IL-1β, and TNF cytokine protein levels were increased, and the same happened at the mRNA level. Altogether, the data show that the main cytokines upregulated are those that play a role in inflammation or neurodegeneration, whilst the main cytokines downregulated were growth factors that may exert neuroprotective activity. The fact that only pro-inflammatory cytokines together with cytokines involved in neurodegeneration are upregulated, reinforces the idea that an inflammatory process is involved in these FRDA patient-derived OE-MSCs. Indeed, it has been shown that Schwann cells [79] and astrocytes [20] produce pro-inflammatory cytokines and undergo a similar degeneration process, suggesting that non-cell autonomous toxicity is a factor to consider in FRDA.

## 4. Materials and Methods

### 4.1. Patients

Olfactory mucosa biopsies for experiments in this study were obtained from human subjects according to the Code of Ethics of the World Medical Association (Declaration of Helsinki) after approval by the Ethics Committee of the Hospital General Universitario Gregorio Marañón, in Madrid, Spain (No. 6/2006, 27/04/2006). Biopsy and cell isolation were based on a previously described protocol [28,29]. Healthy subjects and FRDA patients were enrolled on a voluntary basis, providing a written informed consent to participate in the study and supplying a biopsy that was acquired following the ethics committee recommendations.

### 4.2. Cell Culture and Differentiation Protocol

Human olfactory mucosa stem cells were obtained from biopsies of healthy subjects (C) and FRDA patients (FA). Cells were cultured in cancer stem cell (CSC) medium composed of Dulbecco´s Modified Eagle Medium (DMEM)/F12 medium supplemented with: GlutaMAX, 0.5% Albumax I, 0.5% Hepes 10 mM, 1% N2 (all from Gibco, Barcelona, Spain), 0.6% Glucose (Sigma-Aldrich, Madrid, Spain), 2% foetal bovine serum (FBS), non-essential amino acids (NEAA, L-Ala 44 mM, L-Asn 45 mM, L-Asp 40 mM, L-Glu 40 mM, L-Pro 30 mM), 0.1% penicillin/streptomycin and freshly added 8 ng/mL recombinant human fibroblast growth factor-2 (rhFGF-2, PeproTech, Rocky Hill, NJ, USA), and 50 ng/mL nerve growth factor (NGF, Sigma-Aldrich) [85]. Cells were maintained at 37°C in a humidified atmosphere containing 5% CO2. Confluent cultures were detached with trypsin/EDTA (Gibco) and passaged every 2–3 days (ratio 1:3) to plates pre-coated with Matrigel (BD Biosciences, Bedford, USA). We selected only those cell lines that maintained a consistent growth rate between early (≥10) and late passage number (≤25), performing all experiments with cells that had less than 25 passages, thus ruling out differences in measured parameters due to passage number.

In addition, to attribute the observed changes to the lack of frataxin, as the main difference between cell lines, we plated equal cell numbers at the beginning of all experiments.

The protocol for neuronal differentiation was adapted from a two-step protocol based on HIF-1α activation and the ROCK (Rho-associated kinase) inhibition approach [86]. In the first step, cells were incubated 4 days in Neurobasal Medium supplemented with B27 (all from Invitrogen, Barcelona, Spain), 2 mM glutamine, 2% FBS, 100 μM CoCl2, 30 μM Y-27632 and 2 mM di-butyryl-cAMP (dbcAMP), refreshing the medium every 2 days. All reagents are from Sigma-Aldrich. In the second step, we used the same medium without CoCl2 and adding 50 ng/mL Brain-derived neurotrophic factor (BDNF) (Alomone Labs, Jerusalem, Israel) for 3–4 days.

### 4.3. Lentiviral Production, Titration and Cell Transduction

The lentiviral vector encoding human frataxin cDNA (pLV-Frat) was a kind gift of Alexander and Fleming (The Children’s Hospital at Westmead and Children’s Medical Research Institute, Sydney, Australia) [87]. The production of lentiviral particles was performed using transient transfection of human embryonic kidney 293T cells with Lipofectamine (Invitrogen) and Plus Reagent (Invitrogen), following the manufacturer´s recommendations. Briefly, 5 µg of the lentivector construct were co-transfected with 5 µg of the packaging plasmid, pCMVdR8.74 “Addgene plasmid 22036”, providing all vector proteins driven by the hCMV promoter except the envelope protein, and 2 µg of the plasmid pMD.2G “Addgene plasmid 12259” encoding the heterologous vesicular stomatitis virus envelope, in a p100 plate previously seeded with 2 × 10^6^ cells. Viral supernatant was collected 48 h post-transfection. Lentiviral packaging, stock production and titration was performed as previously described [88].

To perform frataxin rescue experiments, we plated 25,000 cells/cm^2^ of each cell line, adding increasing amounts of viral supernatants, and choosing a multiplicity of infection (MOI) of 10, with which we achieved a transduction efficiency of ~80 % after 24 h in culture.

### 4.4. GAA Repeat Amplification

Genomic DNA from control and FRDA patient cells was extracted using the QIAgen Genomic tips and QIAgen DNA Buffer Set Kit (Qiagen, Madrid, Spain), according to the manufacturer´s protocol. Amplification of GAA repeats was performed as described by Campuzano et al. [5].

### 4.5. RNA Isolation, cDNA Synthesis and PCR

Total RNA was prepared using the RNeasy Mini Kit (Qiagen) according to the manufacturer’s protocol, with additional on-column DNAse digestion (Qiagen). RNA concentration was determined with a Nanodrop ND-1000 spectrophotometer and its integrity checked by electrophoresis in 1% agarose gel. First strand cDNA was synthesized from 3 μg of total RNA using Superscript III reverse transcriptase (Invitrogen) following the manufacturer’s instructions. Amplification was performed in a 25-μL PCR using 1 µL of the resulting cDNA, using the conditions described in Table 2. Primers were designed in house except for Pax3, PTCH1 and TrkB [89] and β-Actin [90].

### 4.6. Quantitative PCR (qPCR)

Total RNA and first strand cDNA were prepared as described above. Real time PCR was conducted with the 7900HT Fast system (Applied Biosystems, Barcelona, Spain) at 95 °C for 20 s, followed by 40 cycles of 1 s at 95 °C, and 20 s at 60 °C. cDNA was amplified using Fast SYBR^®^ Green Master Mix (Applied Biosystems) with the primers detailed in Table 3 (all from Sigma-Aldrich). All primers were tested for their specificity by conventional PCR before being used for the real-time PCR quantitative studies (data not shown). The cycle threshold (Ct) value for each gene was obtained from the real-time PCRs, and the amount of each target mRNA was calculated based on a standard curve and the Ct value. The amount of mRNA for each gene was normalized to β-actin mRNA and presented as relative values.

### 4.7. Cell Lysis and Western Blotting

Cells were washed twice with phosphate-buffered saline (PBS), harvested, placed on ice and then homogenized in a buffer containing: 20 mM Hepes (pH 7.4), 100 mM sodium chloride (NaCl), 100 mM sodium fluoride (NaF), 1% Triton X-100, 1 mM sodium orthovanadate (Na3VO4), 5 mM ethylenediaminetetraacetic acid and the COMPLETE^TM^ protease inhibitor cocktail (Roche Diagnostics). The soluble protein fraction was obtained by centrifugation at 16000× *g* for 10 min at 4 °C. The protein concentration was measured using the Bio-Rad DC protein assay, according to the manufacturer´s instructions (Bio-Rad Laboratories, Hercules, CA, USA). Subsequently, samples were mixed with electrophoresis buffer containing sodium dodecylsulphate (SDS), boiled for 5 min and separated by gel electrophoresis in the presence of SDS on 8–15% acrylamide–bisacrylamide gels. The proteins were then electrotransferred to nitrocellulose membranes (Fischer Scientific, Madrid, Spain) following standard procedures, and the membranes were subsequently incubated in blocking solution composed of 10% non-fat dried milk in PBS plus 0.2% Tween-20 (PBST). The membranes were incubated overnight with primary antibodies diluted in blocking solution at 4 °C and were then rinsed at least three times in PBST. After incubating with the corresponding peroxidase-conjugated secondary antibody for 1 h at room temperature, antibody binding was visualized using an enhanced chemiluminiscence detection system (GE Healthcare) and quantified using an imaging densitometer (GS-710; Bio-Rad Laboratories) and NIH ImageJ (Bethesda, MD, USA) open source software. The absolute values (arbitrary units) from each experimental group were normalized to those obtained for β-actin. The results are presented in arbitrary units expressed as the change relative to their controls that were run simultaneously. The following antibodies were used in Western blot: a polyclonal antiserum against human frataxin (R6.3s, 1:1000) raised against the peptide TLGHPGSLDETTYERLAEETLC (Protein Tools, Madrid, Spain); mouse anti-β-actin (1:1000; Sigma-Aldrich) and anti-Mouse and anti-Rabbit peroxidase-conjugated antibodies (1:5000; Promega, Madison, WI, USA).

### 4.8. Immunocytochemistry

Immunostaining was performed on control and FRDA patient cells cultured on coverslips that were fixed at room temperature for 10 min with PBS containing 2% paraformaldehyde (PFA) followed by another 10 min with PBS 4% PFA. After several washes with PBS, the cells were permeabilized in PBS containing 0.1% Triton X-100 and 1% BSA (PBS-TS). The cells were then incubated overnight at 4 °C with the primary antibody diluted in PBS-TS and after washing with PBS, they were incubated for 1 h at room temperature in PBS-TS with the appropriate Alexa-555-conjugated secondary antibody (1:1000; Invitrogen). For CD133 staining, cells were permeabilized with PBS containing 3% BSA, incubated with the primary antibody for 1 h at 4 °C and with the secondary antibody for 1 h at 37 °C. Following extensive washes, the preparations were stained with 4′-6-diamidine-2-phenylindole (DAPI; 1:5000; Sigma-Aldrich) and after washing, the cells were immediately mounted with Fluoromount-G (Southern Biotech, Birmingham, AL, USA). The labelled preparations were examined using the LSM710 confocal scanning system coupled to an AxioImager.M2 vertical microscope (Zeiss, Oberkochen, Germany). The antibodies used were: Stro-1 (1:100; R&D Systems MAB1038, Minneapolis, MN, USA), Nestin (1:200; Millipore MAB5326, Sigma-Aldrich), CD133 (1:100; Cell Signaling Technology 3663, Danvers, MA, USA), neuron-glial antigen 2 (NG2) (1:200; Chemicon, Temecula, CA, USA), Tuj1 (1:1000; Promega G712A), SMI31 (1:1000; Covance, Princeton, NJ, USA) and phosphorylated MAP1B (1:10; Home-made [91]). As positive control we used either undifferentiated SH-SY5Y cells, the human astrocytoma cell line U-373 MG, the astrocytoma cell line U-87 MG, or primary cells from mouse brain. In the secondary antibody control condition, to check for nonspecific binding of the secondary antibody, the primary antibody was omitted, incubating the cells only with blocking solution.

### 4.9. Reactive Oxygen Species (ROS) Generation

The presence of superoxide anion inside the mitochondria was measured with the MitoSOX Red probe (Fisher Scientific) by flow cytometry, as described previously [92]. The cells were incubated for 30 min at 37 °C with 5 µM MitoSOX Red. After this incubation, the cells were trypsinized and washed twice with Hank’s buffered salt solution containing calcium and magnesium, to finally be resuspended in mitochondria incubation buffer (MIB) containing 68 mM sucrose, 10 mM Hepes (pH 7.4), 70 mM potassium chloride (KCl) and 1 mM ethylene glycol-bis (2-aminoethylether)-N, N, N′, N′-tetra acetic acid [92,93]. The cell suspension was analyzed in a FACScalibur flow cytometer and the data obtained using the Cell Quest program was analyzed with the FlowJo software. Data is presented as the increase in fluorescence intensity measured in FL2. The results are normalized using untreated cells as the reference.

### 4.10. Aconitase Activity Measurements

Total aconitase activity was determined by monitoring the conversion of isocitrate to cis-aconitate, which produces an increase in absorbance at a wavelength of 240 nm. The kit Aconitase Enzyme Activity Microplate assay kit (ab109712, Abcam, Cambridge, UK) was used, following the manufacturer’s recommendations except for some minor modifications to scale-down the protocol.

### 4.11. Cell Viability Assays

Cell viability was assessed by calcein acetoxymethyl ester/propidium iodide (calcein/PI) uptake [94]. With this method, we can quantify the proportion of live/dead cells, as dying cells present disrupted membranes that allow incorporation of PI. Briefly, cells were incubated at 37 °C for 30 min with 2 µM propidium iodide (Sigma-Aldrich) and 1 µM calcein/acetoxymethyl ester (Molecular Probes). Subsequently, cells were visualized by fluorescence microscopy using a Zeiss Axiovert 200 inverted microscope and three randomly selected fields were acquired and analysed per well in at least three independent experiments. Cell death was expressed as the percentage of cells that took up propidium iodide in relation to the total cell number. For H_2_O_2_ treatment, both control and FRDA patient-derived cells were seeded in 6-well plates at a density of 1 × 10^5^ cells/well and grown as previously described. Twenty-four hours prior to determining the viability of all the three cell lines, 100 µm H_2_O_2_ (Sigma-Aldrich) was added.

### 4.12. Human Antibody-Based Protein Arrays

Equivalent numbers of control and FRDA patient-derived cells were cultured in complete CSC medium and, 24 h before performing the experiment, medium was changed to serum-free CSC. Serum free medium was collected and analyzed with the RayBio^®^ Human Cytokine Antibody Array C series 1000 kit (Ray Biotech, Peachtree Corners, GA, USA), which can detect 120 different growth factors/cytokines at a time, according to the manufacturer’s instructions. Briefly, each membrane was placed into the provided eight well tray, and 2 mL of blocking buffer was added and incubated at room temperature for 30 min. Blocking buffer was decanted, and membranes were incubated with 1 mL of media from C3, FA1 and FA6 cells at room temperature for 1–2 h. Samples were decanted and containers were washed 3 times with 2 mL of wash buffer I at room temperature with shaking for 5 min. Membranes were further washed 2 times with 2 mL of wash buffer II at room temperature with shaking for 5 min. Then, 1 mL of diluted primary biotin-conjugated antibody was added to each membrane, and incubated at room temperature for 1–2 h. After washing the membranes two times, 2 mL of 1000 fold diluted HRP-conjugated streptavidin were added to each membrane, and incubated at room temperature for 2 h. Membranes were washed again twice, and placed into the detection buffer. Excess detection reagent was drained off and membranes were covered with a clean piece of plastic sheet. Membranes were then developed using the chemiluminescence imaging system ImageQuant™ LAS 4000 mini (GE Healthcare). After blank subtraction, the data were normalized to the positive controls provided by the manufacturers. The results are expressed as mean fold-change of control cells.

### 4.13. Data Processing and Statistical Analysis

The mean ± standard error of the mean (SEM) of at least three independent experiments are represented in the figures. Statistical comparison of the data sets was performed using Student’s *t*-test or ANOVA, when more than two groups were compared. The differences are presented with their corresponding statistical significance or *p*-value, attributing statistical significance when *p* ≤ 0.05. All statistical analyses were performed using the SPSS program.

## 5. Conclusions

OE-MSCs present a phenotype corresponding to mesenchymal stem cells that are amenable to long-term culture, and FRDA patient-derived OE-MSCs display disease hallmarks such as decreased frataxin expression, reduced aconitase activity, and increased ROS production. Thus, our results confirm the utility of FRDA-derived OE-MSCs as a new in vitro model of the disease, highlighting its potential to study the physiopathology of this disease, with the ultimate goal of designing new therapeutic approaches to treat this condition. Future studies should include more samples from healthy donors and patients to verify and expand the results obtained so far.

## Figures and Tables

**Table 1:** Primers used for q-PCR.

Gene	Forward Primer (5′-3′)	Reverse Primer (5′-3′)	Accession No.
*FXN*	TGGAATGTCAAAAAGCAGAGTG	CCACTCCCAAAGGAGACATC	NM_000144
*HGF*	TCGGGGTAAAGACCTACAGGA	AATGGGGAGAGTTATCGAGGT	NM_000601.4
*IL1α*	ATCAGTACCTCACGGCTGCT	TGGGTATCTCAGGCATCTCC	NM_000575.3
*MCP2*	CCGAGGAGCAGAGAGGTTGAGAAC	CTTGGGACATTGGATGTTGGTGATT	NM_005623.2
*MIF*	ACCGCTCCTACAGCAAGC	CGCGTTCATGTCGTAATAGTTG	NM_002415.1
*IL1β*	AAACAGATGAAGTGCTCCTTCCAGG	CATGGCCACAACAACTGACG	NM_000576.2
*BMP-6*	AACCTGGTGGAGTACGACAAG	TCACCCTCAGGAATCTGGGAT	NM_001718.4
*IGFBP2*	GGTATGAAGGAGCTGGCCGTGTTC	CGCTGCCCGTTCAGAGACATCTTG	NM_000597.2
*IGFBP3*	GCCAGGAAATGCTAGTGAGTCG	GGCAGGGACCATATTCTGTCT	NM_001013398
*MIP-3α*	ACATCAATGCTATCATCTTTCACAC	CCAACCCCAGCAAGGTTCTT	NM_004591.2
*VEGFA*	CTCACCAAGGCCAGCACATA	CCACAGGGGAACGCTCCAG	NM_001171624
*GM-CSF*	AGAGACACTGCTGCTGAGATG	CCAGCAGTCAAAGGGGATGA	NM_000758.3
*G-CSF*	CAGAGCCCCATGAAGCTGAT	GGAAAAGGCCGCTATGGAGT	NM_000759.3
*β-ACTIN*	AACTCCATCATGAAGTGTGACG	GATCCACATCTGCTGGAAGG	NM_001101.3

**Table 2:** Cytokines dysregulated in OE-MSCs.

**Up-Regulated Cytokines**	**Name**
Angiogenesis/Proliferation	ANG, ECGF, KITLG (SCF), TGFB1, VEGFA
Iron regulatory proteins	BMP6
Immunity/Inflammation	ENA-78, ICAM1, ICAM3, IL1α, IL1β, IL6, I-TAC, Lymphotactin (XCL1), MIF, MIP1α (CCL3), MIP3α, NAP-2, SDF (CXCL12), TARC (CCL17), TNF
Neurotropic factors	BDNF, GDNF
Growth factors	G-CSF, GM-CSF, IGF1
Growth factor binding proteins	IGFBP3
**Down-Regulated Cytokines**	**Name**
Angiogenesis/Proliferation	PIGF
Immunity/Inflammation	GRO- α (CXCL1), HCC4 (CCL16), MCP2 (CCL8), NTF4
Growth factors	HGF, FGF
Growth factor binding proteins	IGFBP2

**Table 3:** Primers used for RT-PCR.

Gene	Primers (5′-3′)	Tª Annealing	Extension Time
*KFL4* (NM_004235)	Fw: ACCCGGGGCCCAATTACCCA Rv: AAGGCGAGGTGGTCCGACCT	65 °C	30″
*NANOG* (NM_001297698)	Fw: TGATTTGTGGGCCTGAAGAA Rv: GCATGCAGGACTGCAGAGAT	62 °C	45″
*PAX3* (NM_181459)	Fw: AGCACCCCAATCAGATGAAG Rv: TGTCTGGGTTGGAAGGAATC	61 °C	30″
*PTCH1* (NM_001083602)	Fw: CGCACAGAACTCCACTCAAA Rv: GGGCCAGAAGAAAAACATCA	61 °C	20″
*SOX2* (NM_003106)	Fw: ATGTATCTCCCCGGCGCCGA Rv: TCGGCATCGCGGTTTTTGCG	65 °C	30″
*SOX9* (NM_000346)	Fw: CCGACGAGCAGGAGAAGGGCCTG Rv: TCGCGGAAGTCGATAGGGGGC	65 °C	45″
*TRKB* (NM_001291937)	Fw: ACCCCCATTCGCATCTAAC Rv: CAGAAATGCTTTATGAGCCACA	60 °C	45″
*β-ACTIN* (NM_001101)	Fw: CCACACTGTGCCCATCTACGAGGGGT Rv: AGGGCAGTGATCTCCTTCTGCATCCT	60 °C	30″
